# Primary human nasal epithelial cells: a source of poly (I:C) LMW-induced IL-6 production

**DOI:** 10.1038/s41598-018-29765-0

**Published:** 2018-07-27

**Authors:** Mahnaz Ramezanpour, Harrison Bolt, Alkis James Psaltis, Peter-John Wormald, Sarah Vreugde

**Affiliations:** 1 Department of Surgery - Otorhinolaryngology Head and Neck Surgery, the Queen Elizabeth Hospital, and the University of Adelaide, Adelaide, South Australia Australia; 2 College of Medicine and Public Health, Flinders University, GPO Box 2100, Adelaide, South Australia 5001 Australia

## Abstract

Infection plays a significant role in the relapse of chronic rhinosinusitis (CRS), however, the role of primary human nasal epithelial cells (HNECs) in this process is largely unknown. Here, we determined the effect of Toll-like receptor (TLR) agonists and inflammatory cytokines on mucosal barrier integrity and immune response of HNECs. TLR 1–9 agonists and inflammatory cytokines were applied to submerged and/or air-liquid interface (ALI) cultures of HNECs from CRS patients and controls for 24 hours. Interleukin-6 (IL-6) protein levels were determined by ELISA. Mucosal barrier integrity was measured via Transepithelial Electrical Resistance and passage of fluorescently-labelled dextrans. IL-1β and IFN- γ significantly increased IL-6 production in HNECs derived from CRS patients and controls, however, a dose-dependent effect was observed in CRS-derived HNECs only. Stimulation with Poly (I:C) LMW induced a 15 to 17 fold increase in IL-6 production by HNEC-ALI control cells (p < 0.05) and HNEC-ALI-CRS cells (p = 0.004) whilst a 2.5 fold increase was observed in CRS HNEC submerged cultures. Priming of cells with Poly (I:C) LMW reduced subsequent IL-6 secretion upon stimulation with TLR 2–4 agonists. Poly (I:C) LMW exerts a potent pro-inflammatory effect on HNECs and reduces a subsequent immune activation by TLR agonists.

## Introduction

The sinonasal mucosa has been widely recognised as protecting the host from invasion by harmful environmental toxins and micro-organisms by forming a structural barrier. The epithelial apical junctional complex (AJC) which comprises tight and adherens junctions, is critical to maintain mucosal barrier integrity and epithelial cell polarity. Disruption of AJC proteins leads to mucosal barrier dysfunction and is frequently found in severe chronic inflammatory diseases of the gut, skin and airway^1,2^. The role of the airway mucosa in raising and shaping an immune response to different environmental insults has been extensively described and different model systems have been developed. These include *ex vivo* mucosal explant models that have been shown to have a robust response to bacterial triggers^3,4^. The advantage of such models is that they adequately mimic the *in vivo* situation as they represent the combined immune response of the different immune cell types present within the mucosa to such triggers. The disadvantage is that such explant models are inherently stressed due to lack of adequate oxygen and nutrient supply within the tissue, and are viable only for a limited amount of time (depending on the challenge from 24–72 hours)^3^. Also, the mucosa comprises a range of different cell types known to be critical in orchestrating such responses and a specific role of airway epithelial cells within that process has not been fully elucidated^5,6^. Airway epithelial cell culture models are widely used, as such cells are easy to grow and give consistent results with relatively low variability between experiments. However, airway epithelial cell lines will, in general, not form a functional barrier structure and mucociliary transport system and they do not have a conserved innate immune response machinery, hence any findings on the immune response when such cells are used should be interpreted with caution^7^. Primary human nasal epithelial cells (HNECs) are equipped with innate immune receptors and can respond to a range of environmental insults of microbial^7^ and non-microbial origin^8^, contributing to the immune response to those triggers. HNECs cultured at ALI can differentiate into a ciliated, pseudostratified epithelium that secretes mucus, exerts high Trans Epithelial Electrical Resistance (TEER) (a measure of the epithelial barrier function) and has a functional mucociliary transport system, mimicking the air-facing sinonasal epithelium. HNEC-ALI cultures are well suited to study innate immune responses as well as the effect of different products on the mucosal barrier *in vitro*^9–12^.

It has been previously established that HNECs are better suited than airway epithelial cell lines to study barrier structure and function and immune responses^7^. However, it is not clear whether HNECs grown at ALI have a different response to immune stimulation compared to submerged HNECs, and whether cells derived from patients suffering from chronic airway inflammation respond differently from cells derived from control patients. It is also not known which immune triggers consistently induce immune responses by those cells.

This study compares immune responses of submerged and ALI-grown HNECs derived from patients affected by chronic rhinosinusitis and control patients and defines the immune triggers and conditions needed to induce robust immune activation in those cells.

## Methods

### Human primary nasal epithelial Cells

This study was performed in accordance with guidelines approved by the Human Ethics Committee of the Queen Elizabeth Hospital and the University of Adelaide. All patients gave written informed consent (reference HREC/15/TQEH/132) and all samples obtained were anonymised and coded before use. Nasal brushings were collected from consenting participants and exclusion criteria included active smoking, age less than 18 years and systemic disease. Primary human nasal epithelial cells (HNECs) were harvested from the inferior turbinates by gentle brushing from patients that do not have evidence of CRS (control). HNECs from CRS patients with nasal polyps were harvested by gentle brushing of nasal polyps under endoscopic guidance. Nasal brushings were suspended in Bronchial Epithelial Growth Media (BEGM, CC-3170, Lonza, Walkersvill, MD, USA) which contains (Bovine Pituitary Extract [BPE], Hydrocortisone, human Epidermal Growth Factor [hEGF], Epinephrine, Transferrin, Insulin, Retinoic Acid, Triiodothyronine, Gentamicin/Amphotericin-B, and Bovine Serum Albumin – Fatty Acid Free [BSA-FAF]) and supplemented with 2% Ultroser G (Pall Corporation, Port Washington, NY, USA). Extracted cells were then depleted of monocytes using anti-CD68 (Dako, Glostrup, Denmark) coated culture dishes. HNECs were expanded in routine cell culture conditions of 37 °C humidified air with 5% CO_2_ in collagen coated flasks (Thermo Scientific, Walthman, MA, USA).

### Air Liquid Interface Culture

HNECs were maintained at Air Liquid Interface (ALI), following the Lonza ALI culture method (Lonza, Walkersville, USA) as described previously^11,13^. Briefly, 7 × 10^4^ HNECs were seeded in a volume of 100 µL B-ALI medium which contains (Bovine Pituitary Extract [BPE], Hydrocortisone, human Epidermal Growth Factor [hEGF], Epinephrine, Transferrin, Insulin, Retinoic Acid, Triiodothyronine, Gentamicin/Amphotericin-B, Bovine Serum Albumin – Fatty Acid Free [BSA-FAF] and inducer) into the apical chamber of Transwell plates (BD Biosciences, San Jose, California, USA) and 500 µL of B-ALI growth medium was added to the basal chamber in all wells and incubated for 3–4 days at 37 °C with 5% CO_2_. Then, the apical media was removed and 500 µL B-ALI™ differentiation medium was added to the basal chamber. The cultures were fed every other day by adding B-ALI complete differentiation medium to the basal chamber. HNECs at air liquid interface (HNEC-ALI) were maintained for a minimum of 21 days for development of tight junctions.

### Cytokines and TLR agonists

Cytokines were added to the basal Transwell chamber at the following final concentrations: Tumour Necrosis Factor-α (1 ng/ml, 10 ng/ml, 100 ng/ml, Sigma, Saint Louis, USA), Interferon-γ (1 ng/ml, 10 ng/ml, 100 ng/ml, Sigma, Saint Louis, USA), IL-1β (1 ng/ml, 5 ng/ml, 10 ng/ml Sigma, Saint Louis, USA) IL-17A (50 ng/ml, 100 ng/ml, Gibco, Life Technology, USA), IL-22 (50 ng/ml, 100 ng/ml, Sigma, Saint Louis, USA), and IL-26 (50 ng/ml, 100 ng/ml Abnova Taiwan Corp). TLR agonists were added to the apical and basal Transwell chambers at the following final concentrations: TLR1: Pam3CSK4 (1 µg/ml), TLR2: HKLM (10^8^ cells/ml), TLR3: Poly(I:C) HMW (10 µg/ml), TLR3: Poly (I:C) LMW (10 µg/ml), TLR4: LPS (1 µg/ml), TLR5: Flagellin (1 µg/ml), TLR6: FSL-1 (1 µg/ml), TLR7: Imiquimod (1 µg/ml), TLR8: ssRNA40 (1 µg/ml), TLR9: ODN2006 (5 µM).

### Enzyme-Linked Immunosorbent Assay (ELISA)

Supernatant was collected from the basolateral compartment of treated HNEC-ALI cultures after 24 hours of exposure with inflammatory cytokines. Interleukin-6 (IL-6) protein levels were estimated with an ELISA kit using rat anti-human IL-6 antibodies (BD Biosciences, New Jersey, USA), according to the manufacturer’s instructions. All measurements were performed in duplicate using a FLUOstar OPTIMA plate reader (BMG Labtech, Ortenberg, Germany). The tissue sample concentration was calculated from a standard curve and corrected for protein concentration.

### Transepithelial Electrical Resistance (TEER)

Transepithelial electrical resistance (TEER) was measured by using an EVOM volt-ohmmeter (World Precision Instruments, Sarasota, FL, USA). Briefly, 100 µL of B-ALI medium was added to the apical chamber of ALI cultures to form an electrical circuit across the cell monolayer and into the basal chamber. Cultures were maintained at 37 °C during the measurement period using a heating platform. Only wells displaying baseline resistance readings greater than 700 Ω/cm^2^ were used for the experiments. TLR agonists and control (B-ALI medium for the negative control and 2% Triton × 100 for the positive control) were added to the basal and/or apical chambers of each Transwell and TEER measurements were obtained at time 0 and 24 h.

### Permeability Assay

Paracellular permeability was studied by measuring the apical-to-basolateral flux of FITC- dextran 4 kDa (Sigma, Saint Louis, USA). Briefly, after treating the cells for 24 h, the upper chambers were filled with 3 mg/mL of FITC-dextran and incubated for 2 h at 37 °C. Samples of 40 µL were recovered from the bottom chamber and serially diluted on a 96-well plate (Corning-Costar Corp., Cambridge, United Kingdom (96 wells)), and the fluorescence was measured with a microplate fluorometer (FLUOstar Optima, BMG Labtech, Ortenberg, Germany).

### Immunofluorescence microscopy

Cells were fixed with 2.5% formalin in phosphate-buffered saline (PBS) for 10 min. Fixed samples were permeabilized with 0.1% Triton X-100 in PBS for 15 minutes, blocked for 1 hour with Protein Block (Dako), and incubated with 2 μg/ml rabbit polyconoclonal anti-human TLR3 (#LS-B4866, Sigma, Aldrich, USA), overnight at 4°C. In negative controls, the primary antibody was replaced with PBS. Excess primary antibody was removed, and 2 μg/ml anti-Rabbit CY3 conjugated secondary antibody (Jackson ImmunoResearch Labs Inc., West Grove, PA, USA) was added and incubated for 1 hour at RT. The Membranes were rinsed in TBST, and after the third wash, 200 ng/ml of 4′, 6-diamidino-2-phenylindole (DAPI; Sigma, Aldrich, USA) was added to resolve nuclei. Membranes were transferred to a glass slide and a drop of anti-fade mounting medium (Dako, Glostrup, Denmark) was added before cover-slipping. Samples were visualized by using a LSM700 confocal laser scanning microscope (Zeiss Microscopy, Germany). Slide tissue was prepared as above except tissue were cut in 4 µm sections from CRSwNP patients, deparaffinized and rehydrated. Antigen retrieval was induced at 100 °C for 10 minutes in 10 mmol/L sodium citrate buffer, pH 6.

### Statistical analysis

Data are presented as mean ± SEM. The IL-6 assays where HNECs were grown in submerged cultures were analysed using t-tests and all other analysis was performed using ANOVA, followed by Tukey’s HSD post hoc test using SPSS (version 22). A P value less that 0.05 was considered statistically significant.

## Results

### Dose-dependent effect of Interferon-γ and Interleukin-1β on secreted IL-6 protein levels in HNECs derived from CRS patients

**Figure 1:**
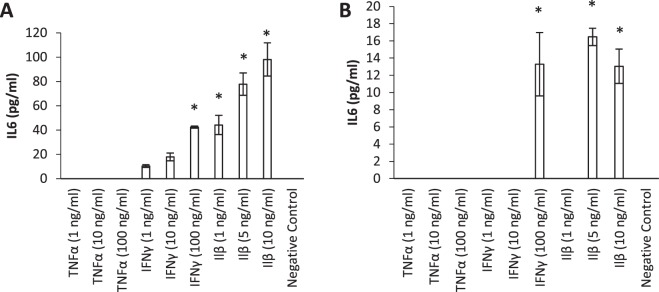
Interleukin-6 secretion by HNEC monolayers derived from CRSwNP patients (**A**) and non-CRS control patients (**B**) in response to inflammatory cytokines. Interleukin-6 protein levels in the basal chamber of HNEC-ALI monolayers from CRSwNP patients (**A**) and non-CRS control patients (**B**) expressed as total protein levels (pg/ml). Cells were exposed to 24 hours of Tumour Necrosis Factor- α (TNF- α) (1, 10, 100 ng/ml), Interferon-γ (IFN-γ) (1, 10, 100 ng/ml), Interleukin-1β (IL-1β) (1, 5, 10 ng/ml) and negative control (medium). The values are shown as mean ± SEM for n = 5 independent donors. Treatments significantly different from the untreated control at P < 0.05 are presented as *; ANOVA, followed by Tukey HSD post hoc test.

Different concentrations of Tumour Necrosis Factor-α (TNF-α), Interferon-γ (IFN-γ), Interleukin-1β (IL-1β) and the Th17 cytokines IL-17, IL-22, and IL-26 were applied to the basal chamber of HNEC-ALI monolayers derived from non-CRS control patients (n = 5, 3 males, 2 females aged 30–50 years) and CRSwNP patients (n = 5, all males aged 45–65 years, 3 were diagnosed with grass-pollen allergy and 4 with asthma) for 24 hours followed by measuring secreted IL-6 protein levels using ELISA. TNF-α and the Th17 cytokines IL-17, IL-22, and IL-26 did not induce IL-6 secretion in any of the HNEC cultures (Fig. 1A,B and Supplementary Fig. S1). Whilst IFN-γ and IL-1β significantly induced the release of IL-6 from both patient groups, IL-6 protein levels were significantly higher upon stimulation with 100 ng/ml IFN- γ (42 pg/ml vs 13 pg/ml in CRSwNP patients and non-CRS controls respectively, P = 0.017) and with 10 ng/ml IL-1β (98 pg/ml vs 13 pg/ml in CRSwNP patients and non-CRS controls respectively, P = 0.025) in monolayers derived from CRSwNP patients, than control patients. Also, IL-1β and IFN-γ increased IL-6 production in a dose-dependent way in HNECs derived from CRSwNP patients but not in non-CRS control derived HNECs (Fig. 1).

### Poly (I: C) LMW increased the secretion of IL-6 from HNECs

**Figure 2:**
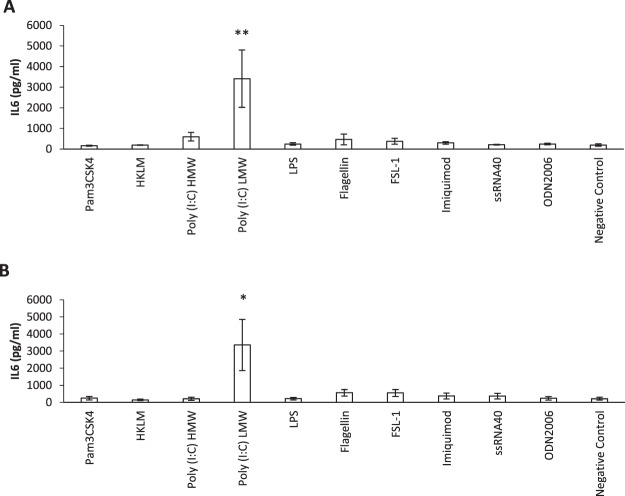
Interleukin-6 secretion of HNEC-ALI monolayers derived from CRSwNP patients and Controls. Pam3CSK4 (1 µg/ml), HKLM (10^8^ cells/ml), Poly (I:C) HMW (10 µg/ml), Poly (I:C) LMW (10 µg/ml), LPS (1 µg/ml), Flagellin (1 µg/ml), FSL-1(1 µg/ml), Imiquimod (1 µg/ml), ssRNA40 (1 µg/ml) and ODN2006 (5 µM) and negative control (medium) were applied on both basal and apical sides in HNEC-ALI cultures from CRSwNP patients (**A**) and non-CRS control patients (**B**) for 24 h. Interleukin-6 expression shown in pg/ml. The values are shown as mean ± SEM for n = 5. *P < 0.05, **P < 0.01. ANOVA, followed by Tukey HSD post hoc test.

**Figure 3:**
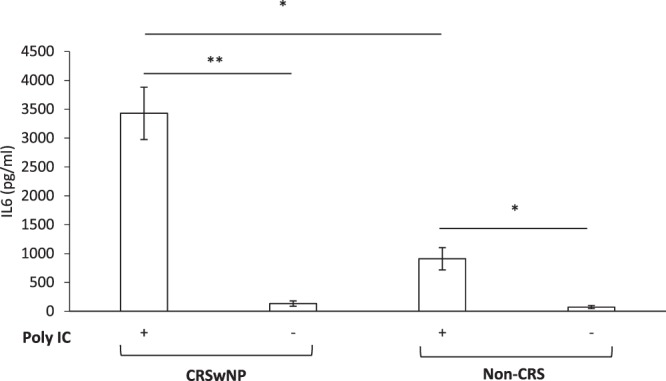
Interleukin-6 secretion by primary human nasal epithelial cells (HNECs) submerged cultures from CRSwNP patients (**A**) and non-CRS control patients (**B**). Interleukin-6 protein levels in the supernatants of HNEC submerged cultures exposed to 24 hours of Poly (I:C) LMW and negative control (medium) expressed as total IL-6 protein levels (pg/ml). The values are shown as means ± SEM, n = 3. *P < 0.05, **P < 0.01, using t-tests.

The effect of TLR 1–9 agonists applied to basal and apical sides of HNEC-ALI cultures on IL-6 secretion was then tested. As shown in Fig. 2A, Poly (I:C) LMW manifestly increased secreted IL-6 protein levels more than 17-fold (3411 pg/ml) compared with negative control (192 pg/ml) in HNEC-ALI monolayers from CRSwNP patients (P = 0.004). Similarly, in non-CRS control patients, Poly (I:C) LMW increased IL-6 over 15-fold (3356 pg/ml) in comparison with negative control (214 pg/ml) (P = 0.012) (Fig. 2B). When CRSwNP-derived HNECs were grown in submerged cultures, IL-6 levels were significantly increased to 3429 pg/ml when treated with Poly (I:C) LMW for 24 h compared with negative control (134 pg/ml) (P < 0.006). Non-CRS control derived HNEC submerged cultures challenged with poly (I:C) increased IL-6 levels to 910 pg/ml compared with negative control (74 pg/ml). IL-6 production by CRSwNP HNEC submerged cultures was significantly higher than IL-6 production from non-CRS control-derived HNECs (P = 0.00003) in the presence of poly (I:C). In the absence of Poly (I:C), IL-6 production by CRSwNP-HNECs was similar to non-CRS control-derived HNECs (P = 0.1) (Fig. 3).

### Poly (I:C) LMW increased the secretion of IL-6 by HNEC-ALI cultures when applied to both apical and basal Transwell chambers

**Figure 4:**
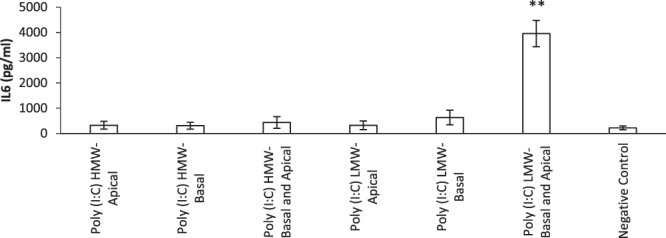
Interleukin-6 production by HNEC monolayers. Poly (I:C) HMW (10 µg/ml), Poly (I:C) LMW (10 µg/ml) or medium (negative control) was applied to the apical, basal or both basal and apical chambers in HNEC- ALI cultures for 24 hours. Interleukin-6 expression shown in pg/ml. The values are shown as means ± SEM, n = 5. Treatments significantly different from the untreated control at P < 0.01 presented as **. ANOVA, followed by Tukey HSD post hoc test.

Poly (I:C) HMW and Poly (I:C) LMW were added to the apical, basal and both apical and basal chambers of Transwells. Poly (I:C) LMW increased the production of IL-6 to 325 pg/ml, 629 pg/ml and 3957 pg/ml when applied in apical, basal and both apical and basal chambers respectively compared with negative control (220.9 pg/ml). The protein level of IL-6 was significantly higher when Poly (I:C) LMW was applied in both basal and apical chambers (p = 0.002). In contrast, Poly (I:C) HMW when applied in apical (322 pg/ml), basal (308 pg/ml) and both apical and basal chambers (435 pg/ml) did not induce any significant increase in the production of IL-6 compared with negative control (220 pg/ml, p > 0.05) (Fig. 4).

### TLR agonists did not affect the permeability of HNEC-ALI monolayers

**Figure 5:**
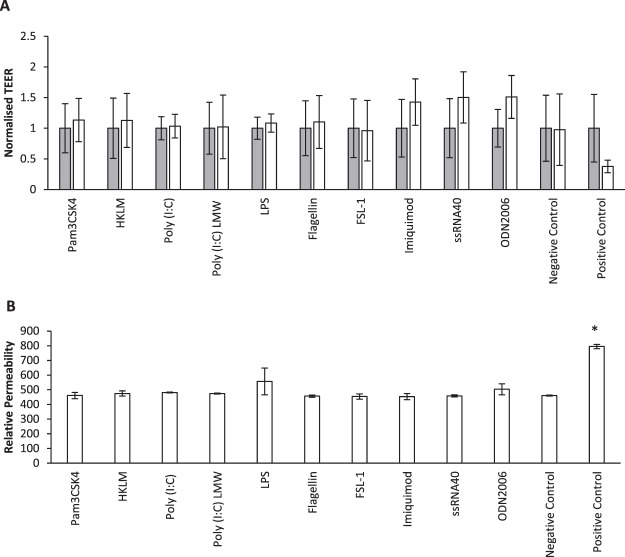
Effect of TLR agonists on Trans-Epithelial Electrical Resistance (TEER) (**A**) and passage of FITC-dextrans (**B**) of HNEC monolayers derived from CRSwNP patients. Effect of Pam3CSK4 (1 µg/ml), HKLM (10^8^ cells/ml), Poly (I:C) HMW (10 µg/ml), Poly (I:C) LMW (10 µg/ml), LPS (1 µg/ml), Flagellin (1 µg/ml), FSL-1(1 µg/ml), Imiquimod (1 µg/ml), ssRNA40 (1 µg/ml) and ODN2006 (5 µM) on the transepithelial electrical resistance (TEER) before the addition of TLRs agonist (time = 0, grey bars), and after 24 h (white bars). Sodium Dodecyl Sulphate (SDS) in B-ALI complete medium was used as a positive control and medium was used as negative control. The values are shown as means ± SEM for n = 5. Treatments significantly different from the untreated control at P < 0.05 presented as *. ANOVA, followed by Tukey HSD post hoc test.

HNEC-ALI cultures were stimulated with TLR1–9 agonists for 24 hours followed by assessment of the permeability of the monolayers by measuring the TEER and passage of FITC-dextrans at 24 h. Sodium Dodecyl Sulphate (SDS) and B-ALI complete medium were used as a positive and negative control respectively. None of the TLR agonists affected TEER measures (Fig. 5A) or permeability of FITC-dextrans (Fig. 5B).

### Priming of HNECs with Poly (I: C) LMW reduces IL-6 secretion after challenge with different TLR agonists

**Figure 6:**
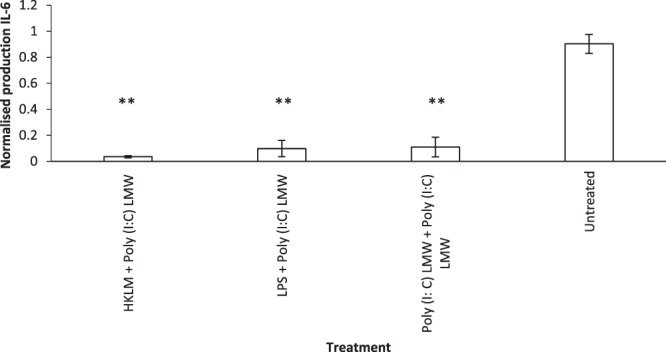
Reduced IL-6 production in response to TLR agonists 2–4 after priming of cells with Poly (I:C) LMW. IL-6 was measured using ELISA after HNEC-ALI cultures were primed with Poly (I:C) LMW (10 µg/ml) then TLR agonist (HKLM (10^8^ cells/ml), LPS (1 µg/ml), or Poly (I:C)) LMW. IL-6 levels were normalised to the non-primed control cells and expressed in pg/mL (n = 5). ANOVA, followed by Tukey HSD post hoc test. **P < 0.001; values are shown as means ± SEM.

To determine whether priming of HNEC-ALI cultures influenced subsequent immune responses, Poly (I:C) LMW was added for 24 h to the basal and apical chambers of HNEC-ALI cultures from control patients. The supernatants were removed and fresh media was added for recovering the cells. After 24 h, the primed cells were treated with the TLR agonists HKLM, LPS or Poly (I:C) LMW. IL-6 secretion was measured in response to TLR agonists in primed and matched non-primed cells and levels were normalised to the non-primed control cells. Primed cells treated with TLR agonists showed lower production of IL-6 protein compared with matched non-primed cells. The results showed that HKLM, LPS 1and Poly (I:C) LMW (p < 0.001) caused significant reduction of IL-6 production after priming of the cells with Poly (I:C) LMW (Fig. 6).

### TLR3 is localised to the sinus epithelium and expressed by HNECs

**Figure 7:**
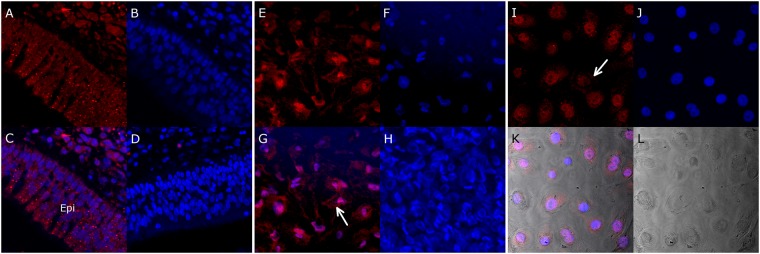
Immunolocalization of TLR3 in sinus mucosa and HNECs. Immunostaining of sinus mucosa (**A**–**D**), HNEC-ALI cultures (**E**–**H**) and HNEC submerged cultures (**I**–**L**). TLR 3 specific staining in red (**A**,**E**,**I**), DAPI staining in blue (**B**,**F**,**J**), overlay (**C**,**G**) and K in whitefield, negative control staining (**D**,**H**) and whitefield in L. TLR immunolocalizes to the epithelial layer (Epi)(**A**,**C**). TLR3 specific staining is seen in the nucleus, cytoplasm and cell periphery (arrows in **G** and **I**).

Immunofluorescence of sinus mucosa, HNEC-ALI and HNEC submerged cultures using TLR3-specific antibodies showed TLR3 expression in the epithelium layer in sinonasal mucosa and in HNECs in the nucleus, cytoplasm and cell periphery (Fig. 7).

## Discussion

Interleukin-6 is known to be produced by airway epithelial cells in response to microbial stimulation and plays a significant role in chronic allergic airway inflammation^3,4,14,15^. This study showed that HNECs derived from CRSwNP patients had a significantly higher IL-6 production compared to HNECs derived from non-CRS control patients in response to a range of immune stimuli including IFN-γ, IL-1β and Poly (I:C) LMW. In addition, IFN-γ and IL-1β increased IL-6 production in a dose-dependent manner in CRSwNP derived HNECs but not in non-CRS control derived HNECs. Previous studies have similarly shown HNECs derived from CRSwNP patients respond differently to topical treatments from HNECs derived from control patients^12^. These donor-dependent differential responses could indicate the presence of genetic alterations and/or epigenetic modifications in CRS patient-derived HNEC cultures that remain active after multiple cell divisions have occurred *in vitro*. Interestingly, recent reports indicate the presence of epigenetic modifications in tissue samples from CRSwNP patients^16,17^, even though the relevance of these findings to CRSwNP-derived HNECs is unclear. Similarly, genetic studies have shown polymorphisms in genes involved in antigen presentation, innate and adaptive immune responses, tissue remodeling and arachidonic acid metabolism in association with CRS (reviewed in^18^). Whether or not (epi) genetic modifications are present in CRSwNP-derived HNECs and whether such modifications might translate to differential innate immune responses of CRSwNP-derived HNECs compared to control-derived HNECs is unknown. Further experiments designed to specifically address these questions are needed to test these hypotheses.

Our results indicate that Poly (I:C) LMW rather than Poly (I:C) HMW consistently induced innate immune activation and IL-6 secretion by HNECs. Both LMW and HMW Poly (I:C) are viral dsRNA surrogates corresponding to viral dsRNA of different lengths (between 0.2–1.0 and 1.5–8 kilobase pairs, for LMW and HMW Poly (I:C) respectively). LMW and HMW Poly (I:C) signal through TLR3 ligation, however, the resulting immune response can differ, with HMW Poly (I:C) inducing more potent TLR3-dependent IFN-β signaling compared to LMW poly (I:C)^19^. In addition to ligating TLR3, LMW Poly (I:C) can also bind Retinoic acid-inducible gene‐I (RIG‐I) whereas HMW Poly (I:C) can bind the related cytosolic helicase, myeloid differentiation-associated gene 5 (MDA5)^20^. The specific structural characteristics of the dsRNA molecules that optimally activate these innate immune receptors are reflected in the types of viruses that are recognized by those receptors. Namely, RIG-I is primarily responsible for innate immune recognition of paramyxoviruses, influenza virus and Japanese encephalitis virus, while MDA5 is thought to be critical for the recognition of picornaviruses^20^. TLR3 on the other hand, recognizes dsRNA molecules greater than 40–50 bp in length and mediates the induction of antiviral responses to, for example, rhinovirus, respiratory syncytial virus and influenza virus, frequently responsible for viral respiratory infections^21^. Our data implies that HNECs might be more sensitive to those viruses that are able to activate TLR3 and/or RIG-I. It is well known that severe bacterial lung infections are often preceded by viral infections^22,23^. Similarly, in the upper airways, viral infections and associated mucosal damage frequently precede bacterial superinfection and acute sinusitis^24^. Moreover, it has been shown that upper airway influenza virus infection significantly alters the sinonasal microbiome and increases the bacterial burden in both upper and lower airways, significantly augmenting the susceptibility to bacterial pneumonia^25^. Our data indicate that priming of HNECs with TLR3 agonists reduces subsequent innate immune responses to a range of TLR agonists. Whilst the mechanism of this finding and its significance remain to be defined, the reduced sensitivity of HNECs to TLR ligation after TLR3 agonist priming might be due to induction of interferon responses. Namely, TLR3 agonists as well as viral infections are known to induce potent interferon responses in a range of immune cell types^26^. Such responses are needed to fight the viral infection. However, they are accompanied by a reduced generalized innate immune response to different immune triggers, facilitating bacterial superinfection^27^. It will be interesting to identify the exact virus types that can stimulate innate immune responses in HNECs and identify and compare their activation and signaling pathways in HNECs.

In conclusion, our data show that HNECs are equipped with innate immune defense mechanisms that allow for a potent immune activation upon ligation of Poly (I:C) LMW and that HNECs derived from CRSwNP patients react more vigorously to immune triggers than HNECs derived from non-CRS control patients. Moreover, we have shown that stimulation with Poly (I:C) LMW reduces subsequent immune activation with different TLR agonists. Together, these data indicate that HNECs play an important role in the immune activation and regulation upon viral infection of the upper airway.

## Electronic supplementary material

Supplementary information

## References

[1] Turner JR (2009). Intestinal mucosal barrier function in health and disease. Nat Rev Immunol.

[2] Weidinger S (2008). Filaggrin mutations, atopic eczema, hay fever, and asthma in children. J Allergy Clin Immunol.

[3] Cantero D (2013). A human nasal explant model to study Staphylococcus aureus biofilm *in vitro*. Int Forum Allergy Rhinol.

[4] Cantero D, Cooksley C, Bassiouni A, Wormald PJ, Vreugde S (2013). Staphylococcus aureus biofilm activates the nucleotide-binding oligomerization domain containing 2 (Nod2) pathway and proinflammatory factors on a human sinonasal explant model. Int Forum Allergy Rhinol.

[5] Hammad H, Lambrecht BN (2011). Dendritic cells and airway epithelial cells at the interface between innate and adaptive immune responses. Allergy.

[6] Ooi EH, Wormald PJ, Tan LW (2008). Innate immunity in the paranasal sinuses: a review of nasal host defenses. Am J Rhinol.

[7] Cooksley C, Roscioli E, Wormald PJ, Vreugde S (2015). TLR response pathways in NuLi-1 cells and primary human nasal epithelial cells. Mol Immunol.

[8] Bardet G (2014). A model of human nasal epithelial cells adapted for direct and repeated exposure to airborne pollutants. Toxicol Lett.

[9] Murphy, J. *et al*. Staphylococcus Aureus V8 protease disrupts the integrity of the airway epithelial barrier and impairs IL-6 production *in vitro*. *Laryngoscope*10.1002/lary.26949 (2017). 10.1002/lary.26949 28994126

[10] Malik Z (2015). Staphylococcus aureus impairs the airway epithelial barrier *in vitro*. Int Forum Allergy Rhinol.

[11] Ramezanpour M, Moraitis S, Smith JL, Wormald PJ, Vreugde S (2016). Th17 Cytokines Disrupt the Airway Mucosal Barrier in Chronic Rhinosinusitis. Mediators Inflamm.

[12] Ramezanpour, M., Murphy, J., Smith, J. L. P., Vreugde, S. & Psaltis, A. J. *In vitro* safety evaluation of human nasal epithelial cell monolayers exposed to carrageenan sinus wash. *Int Forum Allergy Rhinol*10.1002/alr.22021 (2017). 10.1002/alr.22021 29024522

[13] Ramezanpour M, Rayan A, Smith JLP, Vreugde S (2017). The effect of topical treatments for CRS on the sinonasal epithelial barrier. Rhinology.

[14] Rincon M, Irvin CG (2012). Role of IL-6 in asthma and other inflammatory pulmonary diseases. Int J Biol Sci.

[15] Neveu WA (2009). IL-6 is required for airway mucus production induced by inhaled fungal allergens. The Journal of Immunology.

[16] Seiberling KA, Church CA, Herring JL, Sowers LC (2012). Epigenetics of chronic rhinosinusitis and the role of the eosinophil. Int Forum Allergy Rhinol.

[17] Kim JY (2018). Role of epigenetics in the pathogenesis of chronic rhinosinusitis with nasal polyps. Mol Med Rep.

[18] Hsu J (2013). Genetics of chronic rhinosinusitis: state of the field and directions forward. J Allergy Clin Immunol.

[19] Dansako H (2013). Class A scavenger receptor 1 (MSR1) restricts hepatitis C virus replication by mediating toll-like receptor 3 recognition of viral RNAs produced in neighboring cells. PLoS Pathog.

[20] Kato H (2006). Differential roles of MDA5 and RIG-I helicases in the recognition of RNA viruses. Nature.

[21] Vercammen E, Staal J, Beyaert R (2008). Sensing of viral infection and activation of innate immunity by toll-like receptor 3. Clin Microbiol Rev.

[22] Sun K, Metzger DW (2008). Inhibition of pulmonary antibacterial defense by interferon-gamma during recovery from influenza infection. Nat Med.

[23] Iverson AR (2011). Influenza virus primes mice for pneumonia from Staphylococcus aureus. J Infect Dis.

[24] Fokkens, W. J. *et al*. European Position Paper on Rhinosinusitis and Nasal Polyps 2012. *Rhinol Suppl*, 3 p preceding table of contents, 1–298 (2012). 22764607

[25] Planet PJ (2016). Lambda Interferon Restructures the Nasal Microbiome and Increases Susceptibility to Staphylococcus aureus Superinfection. MBio.

[26] Matsumoto M, Seya T (2008). TLR3: interferon induction by double-stranded RNA including poly(I:C). Adv Drug Deliv Rev.

[27] Navarini AA (2006). Increased susceptibility to bacterial superinfection as a consequence of innate antiviral responses. Proc Natl Acad Sci USA.

