# Supplementary material for: Primary human nasal epithelial cells: a source of poly (I:C) LMW-induced IL-6 production
Source: Sci Rep. 2018 Jul 27;8:11325. doi: 10.1038/s41598-018-29765-0 (PMC6063928; doi:10.1038/s41598-018-29765-0)
Supplement: Supplementary file 1 — Supplementary information [file 41598_2018_29765_MOESM1_ESM.pdf]

Primary human nasal epithelial cells: a source of poly (I:C) LMW-induced IL-6 production

Mahnaz Ramezanpour[1], Harrison Bolt[1, 2], Alkis James Psaltis[1], Peter-John Wormald[1], Sarah Vreugde[1*]

1-Department of Surgery - Otorhinolaryngology Head and Neck Surgery, the Queen Elizabeth Hospital, and the University of Adelaide, Adelaide, South Australia.

2-College of Medicine and Public Health, Flinders University, GPO Box 2100, Adelaide, South Australia, 5001, Australia.

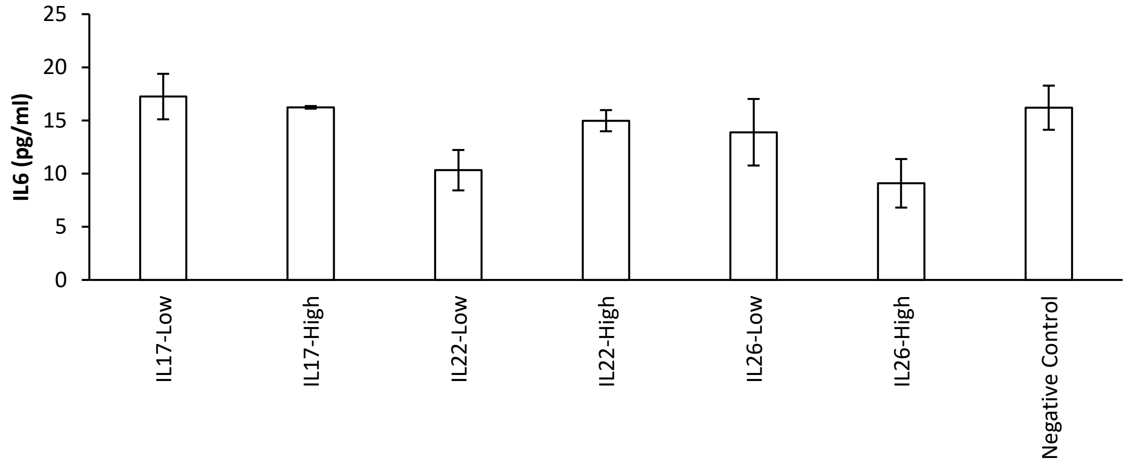

Supplementary Figure S1. Interleukin-6 secretion by HNEC monolayers derived from CRSwNP patients in response to Th17 cytokines. *Interleukin-6 secretion (pg/ml) in the basal chamber of HNEC monolayers in ALI cultures exposed to 24 hours of Interleukin-17 (IL-17), Interleukin-22 (IL-22) and Interleukin-26 (IL-26) at 50 ng/ml (low) and 100 ng/ml (high). Medium was used as negative control. The values are shown as means ± SEM, n = 5.*
